# Camalexin Induces Apoptosis via the ROS-ER Stress-Mitochondrial Apoptosis Pathway in AML Cells

**DOI:** 10.1155/2018/7426950

**Published:** 2018-11-14

**Authors:** Yang Yang, Gang Wang, Wenjun Wu, Shunnan Yao, Xiaoyan Han, Donghua He, Jingsong He, Gaofeng Zheng, Yi Zhao, Zhen Cai, Rui Yu

**Affiliations:** ^1^Bone Marrow Transplantation Center, The First Affiliated Hospital, School of Medicine, Zhejiang University, Hangzhou, 310006 Zhejiang, China; ^2^Department of Biochemistry and Molecular Biology, Zhejiang Key Laboratory of Pathophysiology, Medical School, Ningbo University, Ningbo, 315200 Zhejiang, China

## Abstract

Camalexin is a phytoalexin that accumulates in various cruciferous plants upon exposure to environmental stress and plant pathogens. It was shown that camalexin has potent antitumor properties, but its underlying mechanisms are still elusive. In the present study, we evaluated the effects of camalexin on human leukemic cells and normal polymorph nuclear cells. CCK-8 assay was used to determine cell viability after camalexin treatment. Apoptosis, intracellular reactive oxygen species (ROS) levels, and loss of mitochondrial membrane potential (MMP) were measured by flow cytometry. The activity of SOD, catalase, and ratio of GSH/GSSG were assayed. ER stress and apoptotic signaling pathway was examined by Western blot. Xenograft mice were used to verify the effect of camalexin in vivo. Our results indicated that camalexin inhibited viability of leukemic but not normal polymorph nuclear cells. Furthermore, camalexin induces apoptosis via the mitochondrial pathway in a caspase-dependent manner. We also observed ER stress is located upstream of apoptosis induced by camalexin. Besides, ROS levels, SOD activity, CAT activity, and GSSG levels were significantly enhanced while the GSH level was decreased after treatment of camalexin. In addition, the generation of ROS is critical for the ER stress and apoptosis induced by camalexin. Finally, administration of camalexin suppresses xenograft tumor graft growth without obvious toxicity. Taken together, this study indicates that camalexin exerts antitumor effects against leukemia cells via the ROS-ER stress-mitochondrial apoptosis pathway.

## 1. Introduction

Acute myeloid leukemia (AML), a life-threatening hematological malignancy, is clinically aggressive and characterized by the accumulation of malignant myeloid precursors that halt in differentiation [1], although great advances have been made in the treatment of AML over past years. The 5-year overall survival of adult patients is still unsatisfied, and only one-fifth of elderly AML patients survive more than 2 years [2]. The extremely poor prognosis of AML is largely due to resistance to chemotherapy agents which can also affect normal cells and causing unwanted side effects such as anemia, bleeding, and infection. Therefore, it is urgent to identify novel agents to treat AML.

In recent years, the application of natural products has been widely accepted as an option for the treatment of cancers due to their relatively safety. For example, curcumin, a dietary polyphenolic phytochemical, is able to exert antitumor effects against various cancers [3]. In addition, resveratrol inhibits the proliferation and induces apoptosis in many cancer cells and currently under the evaluation of clinical trials [4]. Camalexin is an indole phytoalexin that accumulates in various cruciferous plants after exposure to environmental stress and plant pathogens. Camalexin has been extensively studied for its role in plant chemical defense mechanisms and has been shown to exert cytotoxic against human protozoan pathogen, *Trypanosoma cruzi* [5]. Camalexin also exhibits antitumor effects against prostate cancer and leukemia cells [6, 7]. However, the underling mechanisms of the antitumor activities of camalexin are still elusive.

In the present study, we examined the antitumor effects of camalexin on primary leukemia cells and leukemia cell lines. Our results showed that camalexin inhibited the viabilities of leukemia cells but not normal polymorph nuclear cells. Our results showed that camalexin induced apoptosis via the ROS-ER stress-mitochondrial pathway in human leukemia cells. Furthermore, in vivo results also indicated that induction of apoptosis may account for camalexin-mediated inhibitory effects on tumor growth of xenograft mouse model. Our findings provide a novel mechanistic basis for camalexin as an agent against leukemia.

## 2. Materials and Methods

### 2.1. Cell Culture

Human AML cell lines HL-60 and NB4 cells were purchased from Shanghai Bank of Cell Culture (Shanghai, China). Fresh leukemia mononuclear cells from peripheral blood of 3 AML patients (FAB classification system) and 2 healthy donors were enriched by Ficoll separation as described before [8]. Informed consent was obtained according to institutional guidelines. Cells were cultured in RPMI-1640 medium (Gibco, NY, USA) supplemented with 10% inactivated FBS (Gibco, NY, USA) and 1% penicillin and streptomycin (Sigma, St. Louis, MO, USA) at 37°C with 5% CO_2_.

### 2.2. Cell Viability Assay

To assess cell viability, cells were seeded in 96-well plates at a density of 1 × 10^4^ cells/well. Then, cells were incubated with various concentrations of camalexin (Sigma, St. Louis, USA) for the indicated times. 10 *μ*l of CCK-8 solution (Sigma) was added to each well followed by incubation for 2 h at 37°C. The cell viability was then assessed by detection of absorbance at 450 nm using a spectrophotometer (BioTek, Winooski, VT, USA).

### 2.3. Apoptosis Assay

Cells were seeded into 96-well plates and incubated with the indicated doses of camalexin. The apoptosis rates were measured by annexin V-FITC apoptosis detection kit (BD Biosciences, San Jose, CA, USA) according to the manufacturer's guide. The apoptosis rates were analyzed by flow cytometry (FACScan, BD, NJ, USA).

### 2.4. Detection of Mitochondrial Membrane Potential (MMP)

Mitochondrial membrane potential (MMP) was assayed using the fluorescent dye JC-1 (Sigma) according to the manufacturer's guide. Briefly, cells were stained with JC-1 for 15 min and rinsed twice with PBS. The concentration of retained JC-1 dye was detected by flow cytometry (BD Biosciences).

### 2.5. ROS Detection

Generation of ROS was measured by 2′,7′-dichlorofluorescin diacetate (DCFH-DA) (Sigma) staining which is converted into fluorescent 2′,7′-dichlorofluorescin (DCF) in the presence of peroxides. Therefore, increase in DCF fluorescent is an indicator of ROS. ROS detection assay kit (Beyotime, Beijing, China) was applied to measure intracellular oxidative stress according to the manufacturer's guide.

### 2.6. Transfection

For knockdown experiments, Bax, PERK, and the negative control (si-NC) shRNA lentiviral were purchased from Sigma. For overexpression of Mcl-1 and catalase, human Mcl-1, catalase, and mitocatalase were synthesized and cloned into the plenti6.3/V5 TOPP vector (Genepharm, Suzhou, China). The 293FT cells were transfected using the ViraPower™ hiPerform™ Lentiviral expression system (Life Technologies). 16 h after transfection, medium was replaced with fresh culture medium. After another 72 h of culture, media were collected and filtered; the supernatant was used for infection.

### 2.7. Measurement of SOD Activity, Catalase Activity, and GSH and GSSG Levels

After treatment with camalexin, the supernatant and cells were collected. The activity of catalase (CAT) and superoxide dismutase (SOD) was assayed by the catalase activity assay kit (ab83464) and superoxide dismutase activity assay kit (ab65354), respectively (Abcam, San Diego, CA, USA). The levels of GSH and GSSG were measured by the GSH and GSSG assay kit (Beyotime, Haimen, China).

### 2.8. Caspase Activity Assay

To detect the activities of caspase-3 and caspase-9, caspase-3 and caspase-9 colorimetric kits were purchased from R&D system. After treatment, the cells were lysed and Ac-DEVD-pNA and Ac-LEHD-pNA were used as caspase-3 and caspase-9 substrates, respectively. Caspase activity and absorbance were measured at OD405.

### 2.9. Western Blot

After treatment, cells were collected and lysed in RIPA buffer. The protein concentrations were measured by Bradford protein assay kit (Sigma-Aldrich). Equal amount of protein was loaded and subjected to SDS-PAGE and transferred to PVDF membrane (Millipore, Boston, MA, USA). After blocking with 5% skimmed milk for 1 h at room temperature, PVDF membrane was incubated with primary antibody overnight at 4°C. The following antibodies were used in our study: anti-caspase-3, anti-caspase-9, anti-Bcl-2, anti-Bcl-xl, anti-Mcl-1, anti-Bid, anti-Bax, anti-cytochrome c, anti-Smac/DIABLO, anti-Bax (6A7), anti-ATF4, and anti-CHOP were purchased from Cellular Signaling Technology (Danvers, MA, USA) and anti-p-PERK, anti-PERK, anti-p-eIF2*α*, and anti-GAPDH (Abcam, San Diego, CA, USA) Then, the membrane was incubated with secondary antibody and visualized by ECL (Thermo Scientific, Rockford, USA). Purification of cytosolic fraction and Bax immunoprecipitation were performed as described earlier [9].

### 2.10. Tumor Xenograft Model

Tumor xenograft model was established to investigate the effect of camalexin in vivo. NB-4 and HL-60 cells were implanted (10^7^ cells/ml) into 6-week-old male BALB/c mice (Wei Tong Li Hua Company, Beijing, China). When the tumor size reached approximate 100 mm^3^, mice were randomly divided into four groups, and treated with intravenous injection of DMSO (vehicle) or different doses of camalexin. Tumors were measured using a caliper every 3 days, and two perpendicular diameters of each tumor were recorded. The tumor volume was calculated in the following formula: volume = (width^2^ × length)/2. Then, tumors were resected and frozen for the Western blot analysis to evaluate the effect of camalexin combination in vivo. All animal experiments followed ethical standards, and all protocols have been approved by the Animal Use and Management Committee of Zhejiang University.

### 2.11. Statistical Analysis

Statistical analysis was performed using the SPSS software (Chicago, IL, USA). Data are expressed as mean ± SD. Differences among groups were tested by one-way ANOVA. A value of *p* < 0.05 was considered as significantly different.

## 3. Results

### 3.1. Camalexin Inhibited Viability of AML Cells but Not Normal Cells

**Figure 1:**
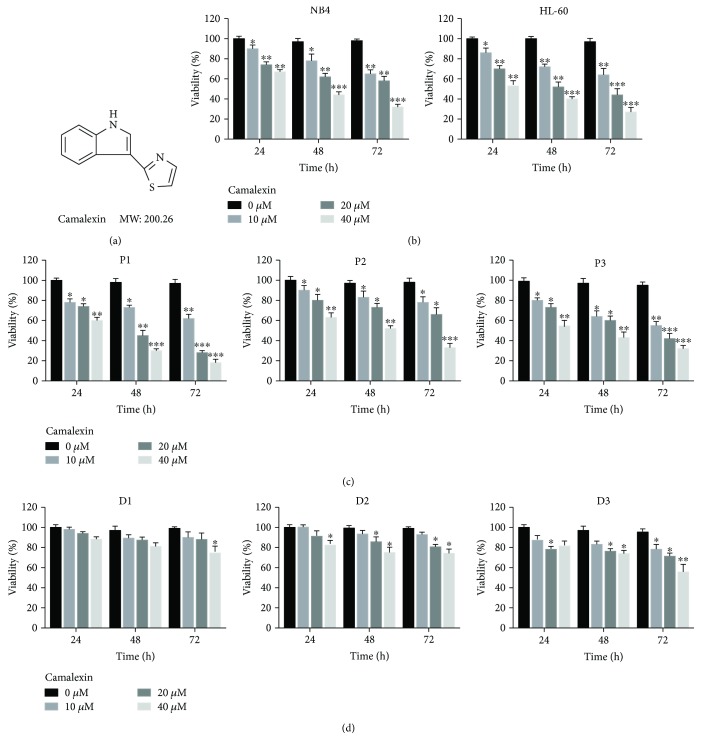
Camalexin decreases viability of human leukemia cells in a dose- and time-dependent manner. (a) The chemical structure of camalexin. (b) NB4 and HL-60 cells were treated with various doses of camalexin (10 *μ*M, 20 *μ*M, 40 *μ*M) for different time (24 h, 48 h, 72 h), and then cell viability was assayed by CCK-8 assay. (c) Primary leukemia cells were isolated from the peripheral blood of 3 patients and treated with various doses of camalexin (10 *μ*M, 20 *μ*M, 40 *μ*M) for different time (24 h, 48 h, 72 h), and then cell viability was assayed by CCK-8 assay. (d) Mononuclear cells were isolated from the peripheral blood of 3 healthy donors and treated with different doses of camalexin (10 *μ*M, 20 *μ*M, 40 *μ*M) for different time (24 h, 48 h, 72 h), and then cell viability was assayed. Mean and SD of three independent experiments performed in triplicate are shown; ^∗^*p* < 0.05, ^∗∗^*p* < 0.01, and ^∗∗∗^*p* < 0.001.

Firstly, we investigated the effects of camalexin (Figure 1(a)) in AML cells. We found that camalexin decreased viability of NB4 and HL-60 cells in a dose- and time-dependent manner (Figure 1(b)). To determine whether camalexin also has effects on primary human leukemia cells, primary leukemia cells isolated from 3 leukemia patients were treated with different doses camalexin for different time. As indicated in Figure 1(c), exposure of cells to camalexin resulted in decreased viability of primary leukemia cell. In contrast, camalexin had little effect on viability of normal bone marrow mononuclear cells (Figure 1(d)). Taken together, these findings suggest that camalexin selectively inhibited viability of primary and transformed human leukemia cells but not normal hematopoietic cells.

### 3.2. Camalexin Induces Apoptosis of Leukemia Cells in a Caspase-Dependent Manner

**Figure 2:**
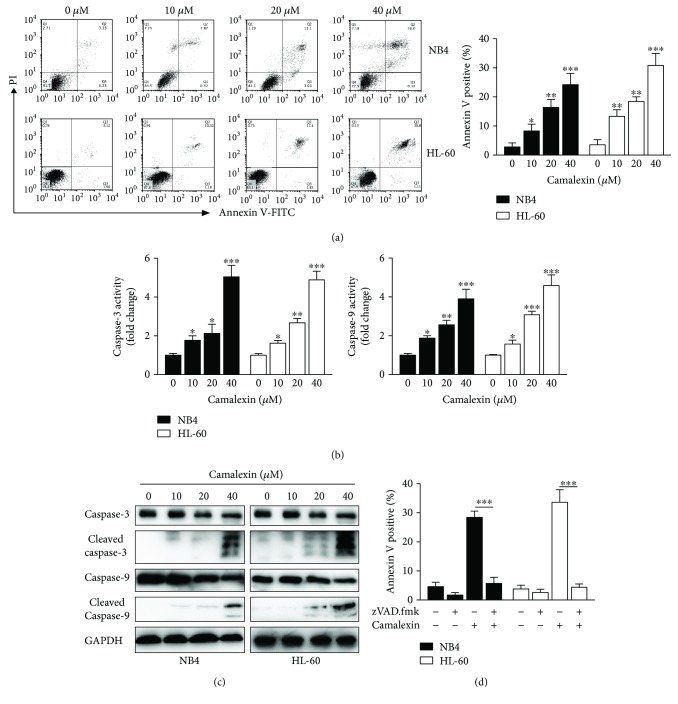
Camalexin induces caspase activation and caspase-dependent apoptosis in leukemia cells. (a, b) Leukemia cells were treated with indicated doses of camalexin for 24 h; cellular apoptosis was measured by annexin V staining and flow cytometry analysis. Caspase activity was assayed by colorimetric assay kit. (c) Leukemia cells were treated with indicated doses of camalexin for 24 h; total cellular lysates were subjected to Western blot analysis with indicated antibodies. (d) Cells were treated with 40 *μ*M of camalexin in the presence or absence of 50 *μ*M zVAD.fmk and apoptosis was assayed. Mean and SD of three independent experiments performed in triplicate are shown; ^∗^*p* < 0.05, ^∗∗^*p* < 0.01, and ^∗∗∗^*p* < 0.001.

To investigate whether apoptosis accounted for the cytotoxicity of camalexin on leukemia cells, flow cytometry analysis was performed and it was shown that camalexin induced apoptosis of NB4 and HL-60 cells in a dose-dependent manner (Figure 2(a)). To elucidate the molecular mechanisms underlying the apoptosis induced by camalexin, caspase activity assays were conducted. As indicated in Figure 2(b), camalexin treatment increased the activity of caspase-3 and caspase-9 in a dose-dependent manner. Consistently, Western blot analysis showed that camalexin treatment leads to the cleavage of caspase-3 and caspase-9 (Figure 2(c)). To explore whether or not activation of caspases is necessary for apoptosis, we added the zVAD.fmk, a pan-caspase inhibitor, to the treatment. As shown in Figure 2(d), zVAD.fmk almost fully blocked apoptosis induced by camalexin. Taken together, these data suggested that apoptosis induced by camalexin depends on activation of caspase.

### 3.3. Camalexin Induces Apoptosis via the Mitochondrial Pathway

**Figure 3:**
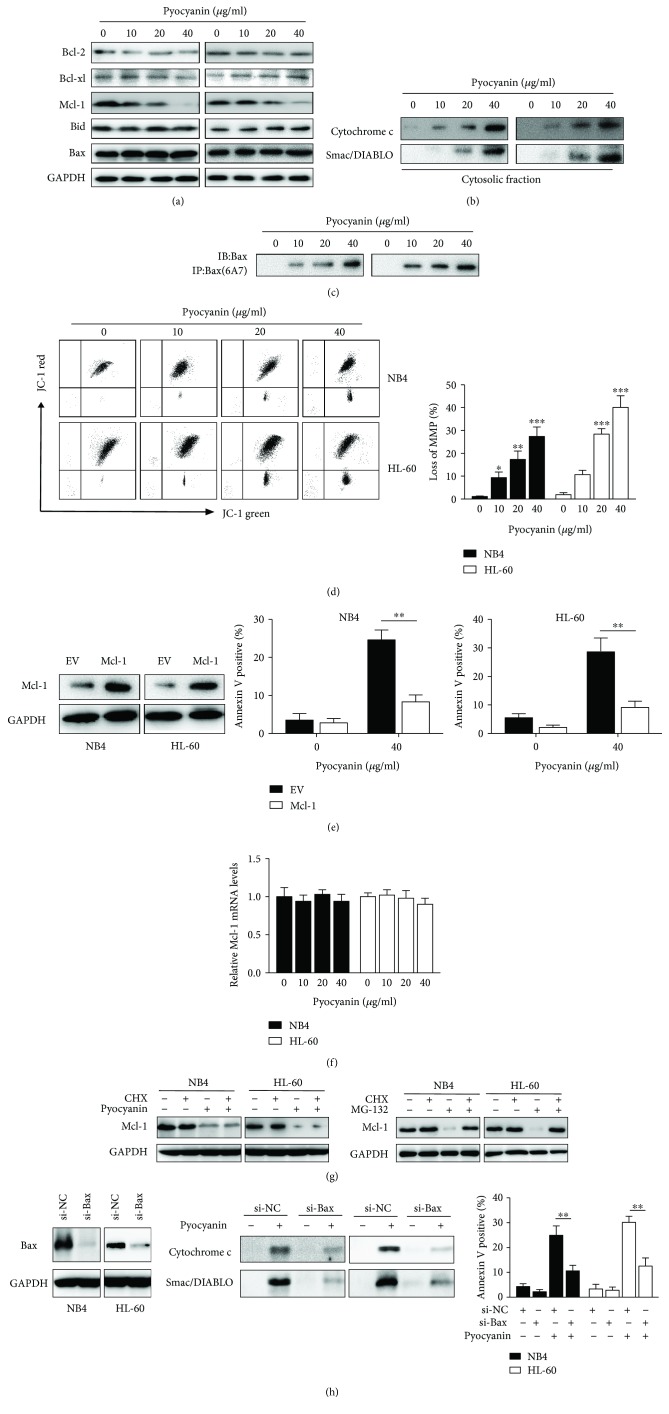
Camalexin triggers the mitochondrial dysfunction pathway. (a, b, c) NB4 and HL-60 cells were treated with indicated doses of camalexin, and then total cellular lysates were subjected to Western blot analysis with indicated antibodies. Activation of Bax was assessed by immunoprecipitation using active conformation-specific antibody. The cytosolic fractions of cells were subjected to Western blot analysis. (d) NB4 and HL-60 cells were exposed to various doses of camalexin for 6 h; disruption of MMP was indicated by the increase of proportion of cells with green fluorescent (left) and decrease in the proportion of cells with higher red (JC-1 aggregates)/green (JC-1 monomers) ratio of JC-1 fluorescent (right). (e) NB4 and HL-60 cells were exposed to various doses of camalexin for 12 h, and then Mcl-1 mRNA levels were evaluated by RT-PCR. (f) NB4 and HL-60 cells were treated with CHX (100 *μ*M) or MG132 (300 nM) in the presence or absence of camalexin (40 *μ*M) for 24 h, and then total cellular lysates were subjected to the Western blotting analysis. (g) Cells were transfected with empty vector (EV) or Mcl-1; the expression of Mcl-1 was analyzed by Western blotting (left). After transfection for 24 h, cells were treated with or without camalexin (40 *μ*M) for another 24 h, and then cellular apoptosis was assayed by flow cytometry (center and right). (h) NB4 and HL-60 cells were transfected with siRNA against Bax for 24 h, and then the expression levels of Bax were evaluated by Western blotting (left). Then, cells were treated with or without camalexin (40 *μ*M) for another 24 h and the release of cytochrome c and Smac/DIABLO into cytosol was measured by Western blot (center). The cellular apoptosis was assayed by flow cytometry (right). Mean and SD of three independent experiments performed in triplicate are shown; ^∗^*p* < 0.05, ^∗∗^*p* < 0.01, and ^∗∗∗^*p* < 0.001.

Since the antiapoptotic Bcl-2 proteins have been described to play essential roles in the process of apoptosis, we then analyzed expression levels of Bcl-2, Mcl-1, Bcl-xl, Bid, and Bax by Western blotting. The expression of Mcl-2 was significantly downregulated after treatment with camalexin (Figure 3(a)). As antiapoptotic Bcl-2 proteins block apoptosis via preventing the activation of the proapoptotic multidomain Bcl-2 protein Bax, the protein level of Bax was not affected after the treatment (Figure 3(a)). We examined whether camalexin treatment results in activation of Bax. To address this question, we immunoprecipitated Bax using conformation-specific antibody that specifically detects the active form. Of note, camalexin treatment leads to the activation of Bax (Figure 3(b)). Since Bax activation leads to the release of mitochondrial proteins into cytosol and permeabilization of mitochondrial outer membrane, we also examined the release of cytochrome c and Smac/DIABLO. As shown in Figure 3(c), the release of mitochondrial proteins cytochrome c and Smac/DIABLO into cytosol was increased by the treatment of camalexin. Moreover, exposure to camalexin caused disruption of MMP as evidenced by an increase in the proportion of cells with green fluorescent light (Figure 3(d)). To examine the effects of camalexin on Mcl-1 downregulation, we measured the mRNA expression of Mcl-1 by RT-PCR and it was shown that camalexin had little effect on the mRNA levels of Mcl-1 (Figure 3(e)). Then, protein synthesis was blocked by cycloheximide (CHX, 100 *μ*M), and the levels of Mcl-2 were examined in the presence or absence of camalexin. As indicated, the Mcl-1 downregulation was not affected by the CHX (Figure 3(f)). In contrast, camalexin failed to repress the expression of Mcl-1 in the presence of a proteasome inhibitor MG132 (Figure 3(f)). Therefore, these findings suggest that camalexin might inhibit Mcl-1 via the accelerated degradation by proteasome. In order to further confirm the role of Mcl-1 in apoptosis induced by camalexin, we overexpressed Mcl-1 and it was revealed that ectopic expression of Mcl-1 significantly decreased the apoptosis induced by (Figure 3(g)). Furthermore, we used siRNA to knockdown Bax, as confirmed by Western blot analysis (Figure 3(h)). Of note, silencing of Bax markedly impaired the release of mitochondrial proteins and apoptosis induced by camalexin (Figure 3(h)).

### 3.4. Camalexin Induces ER Stress in Leukemia Cells

**Figure 4:**
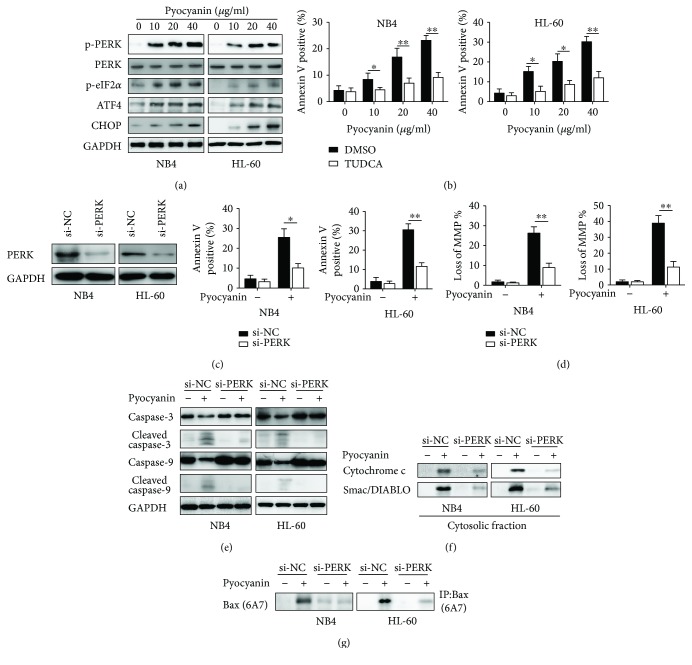
Camalexin induces ER stress in leukemia cells. (a) NB4 and HL-60 cells were treated with various doses of camalexin for 24 h, and then total cellular lysates were subjected to Western blot analysis with indicated antibodies. (b) NB4 and HL-60 cells were treated with various doses of camalexin in the presence of TUDCA (20 *μ*M) or not for 24 h, and then cellular apoptosis was assayed by flow cytometry. (c) NB4 and HL-60 cells were transfected with si-PERK for 24 h, and then PERK levels were evaluated by Western blotting. (d) NB4 and HL-60 cells were transfected with si-PERK for 24 h, and then cells were treated with camalexin (40 *μ*M) for another 24 h and cellular apoptosis was measured by flow cytometry. (e) NB4 and HL-60 cells were transfected with si-PERK for 24 h, and then cells were treated with camalexin (40 *μ*M) for another 24 h and disruption of MMP was measured. (f, g) NB4 and HL-60 cells were transfected with si-PERK for 24 h, and then cells were treated with camalexin (40 *μ*M) for another 24 h, and then total cellular lysates, cytosolic fractions, and activation of Bax were subjected to Western blotting analysis. Mean and SD of three independent experiments performed in triplicate are shown; ^∗^*p* < 0.05 and ^∗∗^*p* < 0.01.

To test whether camalexin also induces ER stress in leukemia cells, we examined the ER stress marker expression after treatment with camalexin in NB4 and HL-60 cells. As indicated in Figure 4(a), camalexin effectively induced the expression of ER stress-related proteins such as phosphorylated PERK (PERK), eIF2a, ATF4, and CHOP in a dose-dependent manner. Previous studies indicated that ER stress might cause mitochondrial dysfunction and related apoptosis [10, 11]. So, we applied TUDCA (tauroursodeoxycholic acid), an ER stress inhibitor, to investigate the relationship between the ER stress and apoptosis induced by camalexin. As shown in Figure 4(b), treatment of TUDCA significantly reduced the apoptosis triggered by camalexin. In order to further confirm the connection between ER stress and apoptosis, siRNA against PERK was applied. After a successful knockdown of PERK (Figure 4(c)), the cellular apoptosis of NB4 and HL-60 cells induced by camalexin was greatly inhibited (Figure 4(d)). Furthermore, the disruption of MMP induced by camalexin was also impaired by silencing of PERK (Figure 4(e)). The apoptosis-related proteins were also examined after knockdown of PERK. Interestingly, it was found that the activation of caspase-3 and caspase-9, release of cytochrome c and Smac/DIABLO, and activation of Bax were blocked by the knockdown of PERK (Figures 4(f) and 4(g)).

### 3.5. Camalexin Induces ROS Generation in Leukemia Cells

**Figure 5:**
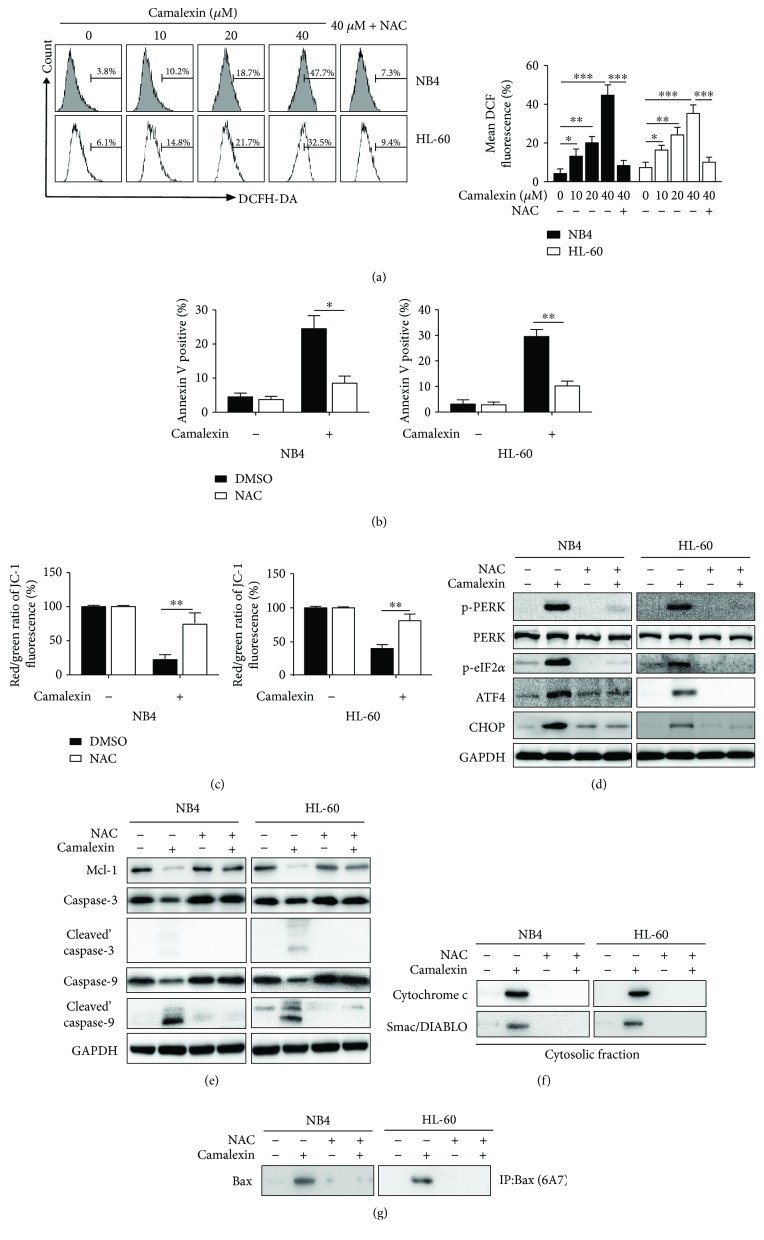
Camalexin induces ROS generation in leukemia cells. (a) NB4 and HL-60 cells were treated with various doses of camalexin for 12 h, and then intracellular ROS was measured by flow cytometry. (b) NB4 and HL-60 cells were pretreated with or without NAC (20 *μ*M) for 4 h, and then cells were incubated with camalexin (40 *μ*M) for 24 h and cellular apoptosis was measured by flow cytometry. (c) NB4 and HL-60 cells were pretreated with or without NAC (20 *μ*M) for 4 h, and then cells were incubated with camalexin (40 *μ*M) for 48 h and disruption of MMP was measured. (d, e) NB4 and HL-60 cells were pretreated with or without NAC (20 *μ*M) for 4 h, and then cells were incubated with camalexin (40 *μ*M) for 24 h and total cellular lysates were subjected to Western blot analysis with indicated antibodies. (f, g) NB4 and HL-60 cells were treated as described above, and then the release of mitochondrial proteins and activation of Bax were assayed by Western blotting. Mean and SD of three independent experiments performed in triplicate are shown; ^∗^*p* < 0.05, ^∗∗^*p* < 0.01, and ^∗∗∗^*p* < 0.001.

Mounting evidence suggests that ROS plays an essential role in ER stress and apoptosis induced by various antitumor agents [12]. Next, we investigated whether intracellular ROS is associated with ER stress and apoptosis induced by camalexin. As shown in Figure 5(a), the intracellular ROS levels were increased in a dose-dependent manner after treatment with camalexin and this effect could be inhibited by ROS scavenger NAC. Then, we used NAC to study the effects of ROS on apoptosis and ER induced by camalexin. It was shown that treatment with NAC significantly impaired the apoptosis (Figure 5(b)) and disruption of MMP (Figure 5(c)) induced by camalexin. Furthermore, the effects of camalexin on ER stress (Figure 5(d)) and Mcl-1 and activation of caspase-3 and caspase-9 (Figure 5(e)) were diminished by NAC. It was shown that NAC could also block the release of cytochrome c and Smac/DIABLO and activation of Bax induced by camalexin (Figures 5(f) and 5(g)). Taken together, these data suggest that ROS generation is essential for the ER stress and apoptosis induced by camalexin.

### 3.6. Oxidative Stress Is Critical for the Apoptosis Induced by Camalexin

**Figure 6:**
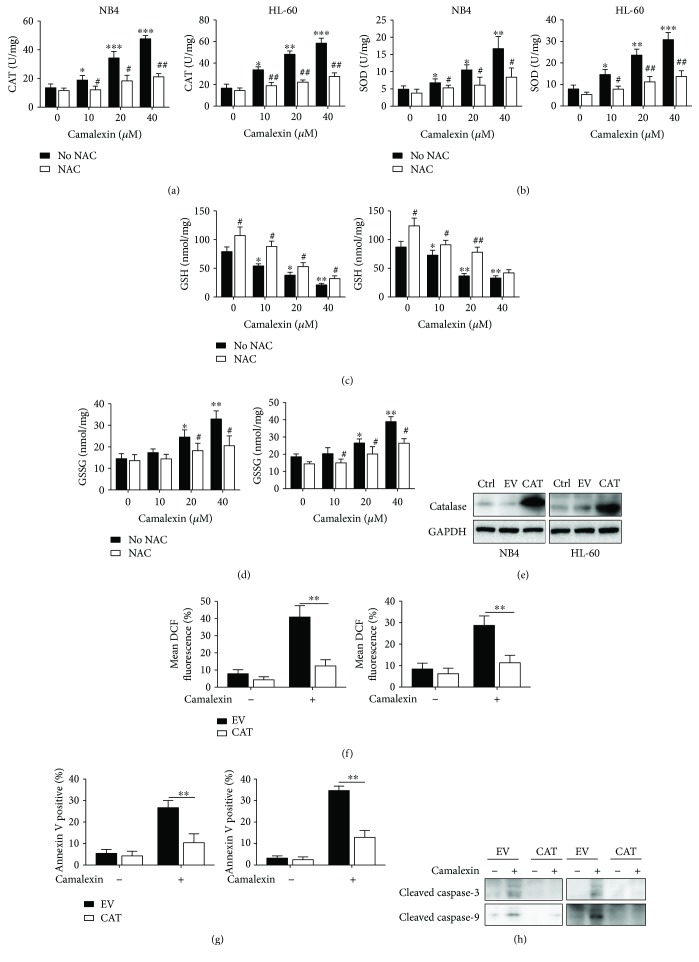
Camalexin-induced oxidative stress is essential for apoptosis. (a, b, c, d) NB4 and HL-60 cells were treated with various doses of camalexin with or without NAC (20 *μ*M), and then the activities of CAT and SOD and the levels of GSH and GSSG were assayed. (e) NB4 and HL-60 cells were transfected with empty vector (EV) or human catalase (CAT) for 24 h, and then the protein levels of CAT were measured by Western blotting. (f) NB4 and HL-60 cells were transfected with empty vector (EV) or human catalase (CAT) for 24 h, and then cells were exposed to camalexin (40 *μ*M) for 12 h, and then cellular ROS levels were measured by flow cytometry. (g, h) NB4 and HL-60 cells were transfected with empty vector (EV) or human catalase (CAT) for 24 h, and then cells were exposed to camalexin (40 *μ*M) for 24 h, and then cellular apoptosis was measured and lysates were subjected to Western blotting analysis with indicated antibodies. Mean and SD of three independent experiments performed in triplicate are shown; ^∗^, #*p* < 0.05, ^∗∗^, ##*p* < 0.01, and ^∗∗∗^*p* < 0.001.

Since cellular damage caused by ROS not only depends on the intracellular levels of ROS but also relies on the balance between ROS and endogenous antioxidant, so we measured the activities of catalase (CAT) and superoxide dismutase (SOD) and increased activities of both CAT and SOD were observed after treatment of camalexin (Figures 6(a) and 6(b)). Meanwhile, the levels of glutathione (GSH) were decreased and the levels of glutathione disulfide (GSSG) were increased after treatment of camalexin (Figures 6(c) and 6(d)). These data demonstrated that exposure to camalexin induced oxidative stress in leukemia cells. In order to further investigate the role of oxidative stress in apoptosis induced by camalexin, we transfected cells with lentivirus containing human catalase (CAT). After transfection for 24 h, the levels of catalase were successfully upregulated (Figure 6(e)). Then, cells were exposure to camalexin (40 *μ*M) for another 12 h and the intracellular ROS levels were significantly reduced after overexpression of CAT (Figure 6(f)). Moreover, annexin V staining showed that the apoptosis-induced camalexin was also diminished by overexpression of CAT (Figure 6(g)). Consistently, the activation of caspase-3 and caspase-9 was also significantly impaired by overexpression of CAT (Figure 6(h)). These results indicate that camalexin-induced apoptosis is due to oxidative stress.

### 3.7. The Antitumor Effects of Camalexin In Vivo

**Figure 7:**
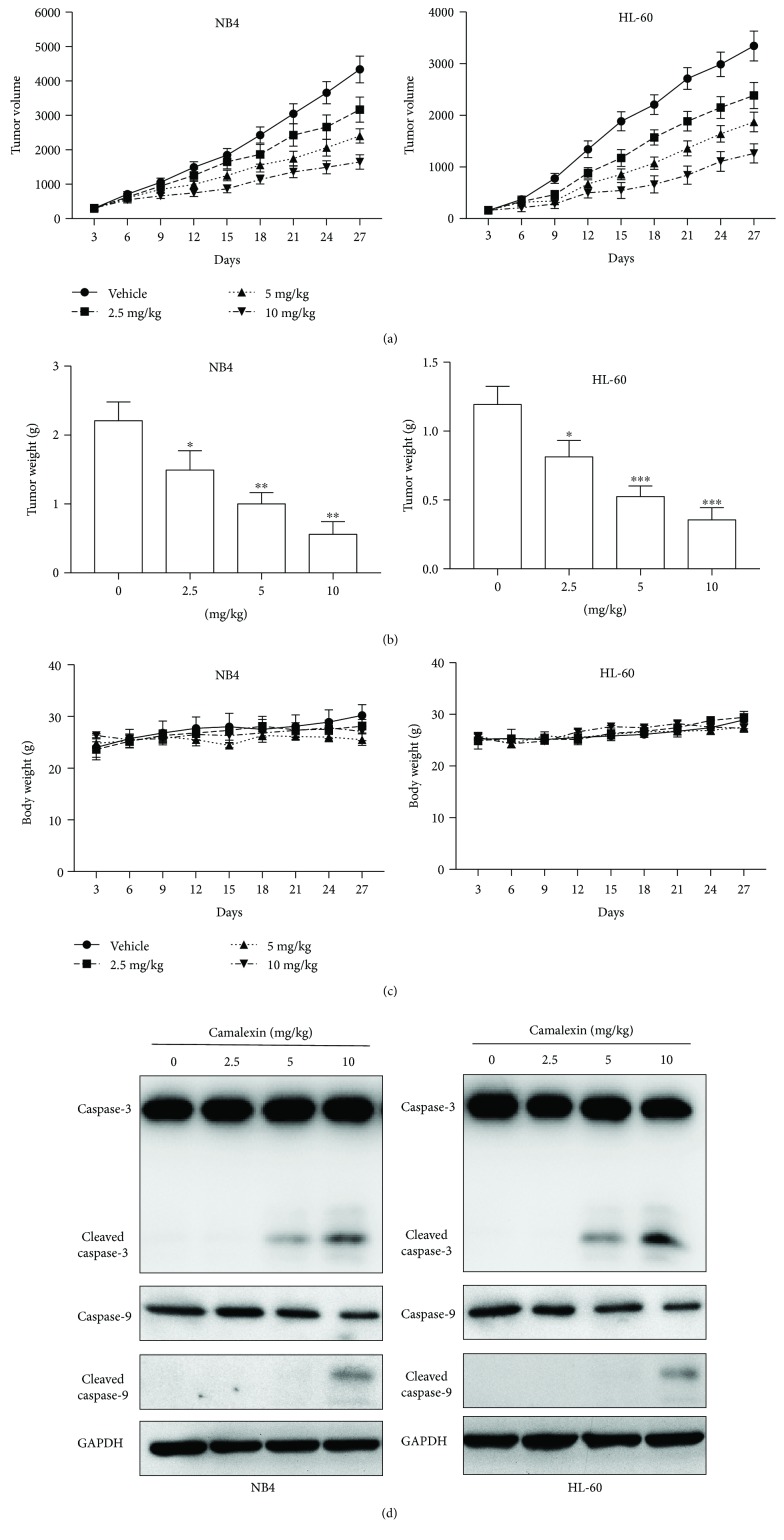
The effect of camalexin on NB4 and HL-60 tumor xenograft. BALB/c mice were ectopically implanted with NB4 or HL-60 cells, and when the tumor size reached around 100 mm^3^, mice were injected with camalexin (i.p.) every three days for 27 days. (a) Effects of camalexin on tumor volume. (b) At day 27, the mice were sacrificed and the tumor weight was measured. (c) Effects of camalexin on body weight. (d) Tumors were resected for Western blot analysis. Data were presented as mean ± SD. ^∗^*p* < 0.05, ^∗∗^*p* < 0.01, and ^∗∗∗^*p* < 0.001.

To evaluate the antitumor effects of camalexin that could be clinically relevant, the antitumor activity of camalexin was evaluated in male BALB/c mice bearing established NB4 or HL-60 tumor xenografts. Mice were randomized into four groups (vehicle, 2.5 mg/kg, 5 mg/kg, 10 mg/kg) and received treatments every five days. As indicated in Figures 7(a) and 7(b), mice treated with camalexin appeared with reduction of tumor volume and weight. Notably, the mice tolerated all of the treatments with no significant body weight difference was observed, indicating that camalexin can be well tolerated (Figure 7(c)). Furthermore, the intratumoral biomarkers were assessed by Western blotting. Consistent with in vitro results, the administration of camalexin significantly induced caspase-3 and caspase-9 activation in tumors, indicating elevated apoptosis (Figure 7(d)). Taken together, these findings suggest that camalexin exerts antitumor effects via induction of apoptosis in vivo.

## 4. Discussion

In the present study, we demonstrate that camalexin, an indole phytoalexin, shows toxicity towards human leukemia cell lines and primary human AML cells. Interestingly, camalexin has little toxicity on normal human peripheral blood monuclear cells, indicating it may serve as a potential agent for cancer chemotherapy.

By using two AML cell lines, we show that camalexin induces apoptosis dependent on the activation of caspases, suggesting the anti-AML activity of camalexin. This is consistent with the previous work on the antileukemia activity of camalexin using Jurkat cell lines [7]. Mezencev et al. detected activation of caspase-8 and caspase-9 after treatment of camalexin. However, we observed activation of caspase-3 and caspase-9 but not caspase-8 (data not shown). This discrepancy may be due to different cell types. Previous studies have indicated that changing expression of Bcl-2 family proteins is associated with the apoptosis induced by various chemotherapeutic agents [13]. Although the levels of Bcl-2, Bcl-xl, Bid, and Bax remained relatively constant, there was significant reduction in the level of Mcl-1 after treatment of camalexin. The augmentation of proteasome activity plays an essential role in the process of protein degradation. In our study, we found that proteasome inhibitor MG132 could inhibit the downregulation of Mcl-1 induced by camalexin. This finding is in line with previous studies in which Mcl-1 degradation is associated with enhanced proteasome activity [14, 15]. Moreover, we also observed activation of Bax and release of mitochondrial proteins into cytosol. These findings suggest that camalexin induced apoptosis via the mitochondrial pathway in leukemia cells.

The ER plays multiple essential roles in regulating various cellular functions, such as proper protein folding, protein synthesis, and calcium homeostasis [16]. In the presence of stress, ER can react in various ways, including the induction of the unfolded protein response (UPR) and apoptosis [17]. In our study, multiple ER stress markers such as phos-PERK, phos-eIF2*α*, ATF4, and CHOP were significantly upregulated after exposure to camalexin. The PERK-eIF2*α*-ATF4-CHOP pathway is a dominant apoptotic signaling pathway triggered by prolonged ER stress [18]. Application of ER stress inhibitor and siRNA against PERK could impede the apoptosis induced by camalexin. Importantly, silencing of PERK also impairs disruption of MMP, release of mitochondrial proteins, and activation of Bax. These data suggest that ER stress is responsible for the mitochondrial apoptosis induced by camalexin. Our findings are in line with previous reports which also showed ER stress could trigger dysfunction of mitochondria and apoptosis [19, 20].

ROS plays a crucial role in the progression of tumors. Tumor cells normally have higher levels of ROS compared to normal cells [21]. Thereby, tumor cells are vulnerable to ROS due to its excessive oxidative stress [22]. The effects of ROS on the development of cancer are complex. It was reported that ROS has the ability to induce DNA mutations and prooncogenic signaling pathway and thereby promote tumor formation [22]. ROS might also directly affect the cellular processes such as proliferation and/or survival [23]. Therefore, abnormal ROS accumulation might be applied as a useful strategy against tumor cells which are more sensitive to ROS accumulation [22]. Multiple agents such as artesunate, NPI-0052, and plumbagin showed toxicity against leukemia cells which are relied on its ability to generate ROS [24–26]. According to previous studies, camalexin could induce ROS in prostate cancer and leukemia cells [6, 7]. Interestingly, camalexin was found unable to induce ROS in erythrocytes in a recent study [27]. This discrepancy may be due to different cell types. We hypothesize that camalexin might possess ability to induce ROS in tumor cells but not normal cells. It could explain the findings in our study that camalexin showed toxicity toward leukemia cells while spares the normal ones and further investigation is required to test this.

To date, little is known about the inhibitory effect of camalexin on tumor growth of xenograft model. The results from in vivo studies demonstrated that camalexin administration significantly repressed the tumor growth of both NB4 and HL-60 xenografts without obvious side effects.

## 5. Conclusion

In summary, our studies indicate that camalexin effectively inhibits viability of human primary leukemia cells and leukemia cell lines, as well as in leukemia xenografts. This effect occurs in association with the activation of ER stress and mitochondrial apoptosis which are relied on the generation of ROS. The antileukemia activity of camalexin found both in vitro and in vivo makes it a potential antitumor agent for hematologic malignancies. It would be intriguing to test camalexin alone or in combination with other chemotherapeutic agents to treat leukemia clinically.

## Data Availability

The data used to support the findings of this study are available from the corresponding author upon request.
